# The Neural Distribution of Vasotocin, Oxytocin, Dopamine, and Serotonin in Two Australian Skinks With Contrasting Social Lives

**DOI:** 10.1002/cne.70184

**Published:** 2026-07-20

**Authors:** Jai Lake, Martin J. Whiting, Geoffrey M. While, David Kabelik, Dustin R. Rubenstein, Daniel Hoops

**Affiliations:** ^1^ School of Natural Sciences Macquarie University Sydney New South Wales Australia; ^2^ School of Natural Sciences University of Tasmania Hobart Tasmania Australia; ^3^ Department of Biological Sciences Thompson Rivers University Kamloops British Columbia Canada; ^4^ Department of Ecology, Evolution and Environmental Biology Columbia University New York City New York USA

**Keywords:** dopamine, lizard, oxytocin, serotonin, social behavior, vasotocin

## Abstract

The neurotransmitters vasotocin, oxytocin, dopamine, and serotonin are widely involved in vertebrate social behavior, and changes in their abundance and distribution in the brain have been linked to the evolution of complex sociality. Reptiles provide an excellent system in which to investigate the neural mechanisms of social living. Using immunohistochemistry, we compare distributions of these transmitters in two skinks differing primarily in social ecology: the family‐living *Liopholis whitii* and the solitary *Eulpamrus quoyii*. We describe patterns of immunopositive signal for both cell bodies and fibers across the entire brain (excluding the olfactory bulbs). In both species, vasotocin and oxytocin were found in the preoptic area, paraventricular nucleus, supraoptic nucleus, dorsomedial hypothalamus, and supraoptic decussation, as well as surrounding the lateral forebrain bundle. Tyrosine hydroxylase (a marker for dopamine) was found in the paraventricular organ nucleus, substantia nigra, and ventral tegmental area, and serotonin was found in the raphe nuclei and superior reticular field. We found novel oxytocin cell groups in the dorsomedial hypothalamus and cerebellum of *L. whitii*, and novel serotonin signal in the red nucleus of *E. quoyii*. Immunopositive signals found only in *L. whitii* also include vasotocin in the ventral tegmental area, tyrosine hydroxylase in the interpeduncular nucleus, and serotonin in the suprachiasmatic nucleus. The qualitatively greater abundance of these transmitters in the family‐living *L. whitii* suggests that these molecules may have played an important role in the evolution of social behavior in these skinks and provides a foundation for broader comparisons across the social skinks.

## Graphical abstract

1. We investigated two skink species differing primarily in social ecology. 2. Using immunohistochemistry, we investigated the distribution of four neurotransmitters known to play a role in vertebrate social behavior. 3. We generally found these transmitters in more brain regions in the family‐living species.

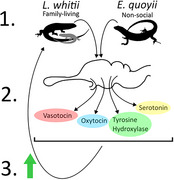

## 1 Introduction

The neurotransmitters vasotocin (VT), oxytocin (OT), dopamine (DA), and serotonin are widely involved in vertebrate social behavior (Adkins‐Regan 2009) and variability in the distribution of these transmitters and their receptors is linked to varying social and reproductive strategies (Goodson et al. 2006; Olazábal and Sandberg 2020; Raghanti et al. 2018). Mapping the distribution of these neurotransmitters and comparing them between species differing in social behavior is a good starting point for understanding the neural changes that accompany and may have facilitated an evolutionary shift toward social living.

While all four neurotransmitters have been shown to be involved in social behavior, the specific social context within which each play a role differs depending on the neurotransmitter. For example, OT and VT play key roles in mediating courtship behavior, pair bonding, mate choice, parental care, social recognition, and aggression (see Donaldson and Young 2008; Insel 2010; Rigney et al. 2022 for reviews). DA is heavily involved with reward‐seeking behavior in mammals, through mediating conditioned preferences for stimuli such as food, water, or sexual contact (reviewed in Wise 2004). In highly social species, DA's role in learning and memory has also been co‐opted to help facilitate parental care and even monogamy (Curtis et al. 2006). Finally, serotonin (5‐hydroxytryptamine [5‐HT]) has been shown to mediate behaviors such as risk assessment, appetite, cooperation, anxiety, sleep, impulsivity, and learning in a wide range of vertebrate and invertebrate taxa (reviewed in Bacqué‐Cazenave et al. 2020).

The majority of work showing how OT, VT, DA, and 5‐HT act as the proximate moderators of social behavior has focused on species with long evolutionary histories of sociality, such as birds and mammals. In contrast, less is known about the role these neurotransmitters play in mediating social behavior in species with less complex and derived social systems, such as non‐avian reptiles (reptiles hereafter). Reptiles have traditionally been overlooked in the study of social evolution, but the diversity of social systems and behaviors displayed (Doody et al. 2021; Whiting and While 2017), together with their phylogenetic position, makes them excellent models for investigating the neural systems underpinning early transitions to sociality and assessing the extent to which these systems converge with those underlying more complex forms of social life (Kabelik and Hofmann 2018; Naumann et al. 2015). The Australian social skinks (tribe Tiliquini) are particularly good models in this context (Van Dyke et al. 2021). They exhibit diverse social systems across closely related species, spanning species that are largely solitary, to species that form facultative associations between parents and offspring, and to species that live in large stable family groups with overlapping generations (Chapple 2003; Whiting and While 2017, 2026).

Work carried out thus far suggests that these four neurotransmitters fulfill similar roles in reptilian social behavior as they do in other vertebrates. For example, artificially blocking VT disrupts the maternal behavior of rattlesnakes (Lind et al. 2017) and increased VT activity correlates with lower aggression in anoles (Kabelik et al. 2022). OT (Ile^8^–OT, also called mesotocin in reptiles) has been shown to be important in the activation and timing of nesting behavior in turtles (Carr et al. 2008), and one study has found that OT‐producing cells in the brain are active in anoles during courtship behaviors (Kabelik and Magruder 2014). DA receptor stimulation has also been shown to reduce aggressive behaviors in lizards (Höglund et al. 2005; Smith and Kabelik 2017). Further, reproductive behaviors are modulated by DA; in two species of whiptail lizards, activation of DA D1 receptors via an artificial agonist increased mounting behavior (Woolley et al. 2001) and in anoles, activation of the D2 receptor inhibited sexual behaviors (Smith and Kabelik 2017). Finally, anoles have also been used to test the social role of 5‐HT. For example, experimentally increasing 5‐HT can lower aggression and cause a dominant individual to become subordinate (Deckel 1996; Larson and Summers 2001). Courtship and aggressive behaviors are both tied to a decrease in serotonergic activity in some brain regions, but increased 5‐HT is linked to initiating these behaviors in other regions (Hartline et al. 2017).

**FIGURE 1:**
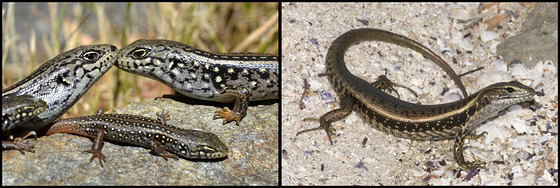
Left: White's skink (*Liophoplis whitii*), a facultatively family‐living species. Right: Eastern water skink (*Eulamprus quoyii*), a predominately solitary species (photos by Geoff While and Martin Whiting).

Here we describe the distribution of VT, OT, tyrosine hydroxylase (TH) (a marker for DA), and 5‐HT in two Australian skink species: the White's skink (*Liopholis whitii*), a facultatively family‐living member of the Tiliquini, and the eastern water skink (*Eulamprus quoyii*), a much less social species (Figure 1). Both are medium‐sized, primarily insectivorous, diurnal, and viviparous. *L. whitii* are found in nearly all habitats in southeastern Australia from sea level to 1600 m (Wilson and Swan 2020). They generally live in small stable family groups characterized by high levels of genetic monogamy, delayed dispersal of offspring, and high levels of sibling conflict (Botterill‐James et al. 2017; Chapple and Keogh 2006; While et al. 2009). *E. quoyii* is also geographically widespread but does not live in family groups (Wilson and Swan 2020), with juveniles typically dispersing to low‐quality marginal habitat after birth (Law 1991). These two species, differing most notably in social ecology, provide an excellent opportunity to examine the neural structures underlying the evolutionary transition to social behavior.

## 2 Materials and Methods

### 2.1 Husbandry and Sample Collection

We caught gravid female *L. whitii* (*n* = 4) in the austral summer of 2021–2022 in Tasmania, Australia. Lizards were transported back to the University of Tasmania, housed in individual plastic terraria (60 × 40 × 30 cm) in a room illuminated with overhead heat and UV lights (10% UVB, ReptiGlo) on a 14:10 cycle to replicate conditions experienced in the wild. Each terrarium was also fitted with a 25 W basking light on a 10:14‐h cycle. Lizards had ad libitum access to water and were fed three times a week on mealworms (*Tenebrio molitor*) and baby food (Heinz, fruit or meat purée). After giving birth, females were housed with one of their offspring for 8 weeks, after which time they were euthanized.

We caught gravid female *E. quoyii* (*n* = 3) in the austral summer of 2020–2021 on Macquarie University Wallumatagal Campus, Sydney, Australia. Lizards were housed in individual plastic terraria (67 × 45 × 38.5 cm), illuminated with overhead 30 W UV light (10% UVB, Ultimate Reptile Suppliers) and fitted with a heat cord underneath one end to provide a temperature gradient (22°C–32°C). Heating and lighting were set to a 12‐h cycle starting at 07:00 h to simulate natural conditions. Lizards were fed three times a week on crickets (*Acheta domesticus*) and were provided with water ad libitum, in a dish large enough for them to completely submerge themselves. Following birth, the mothers were euthanized.

The offspring were not used for this experiment. The difference in the timing of euthanasia of the mothers was due to the demands of separate behavioral experiments the females were involved in. Euthanasia was achieved via a lethal dose (100 mg/kg) of sodium pentobarbitone. Brains were then fixed by immersion in 4% paraformaldehyde (PFA) weight‐by‐volume dissolved in 1× phosphate buffered saline (PBS) overnight at 4°C. The following day, we extracted the brains and postfixed them in 4% PFA at 4°C overnight. For details on the lizard brain fixation protocol we used, see Hoops (2015). Brains were then moved into a liquid cryoprotectant solution (20% glycerol and 30% ethylene glycol in 1× PBS) and stored at −20°C. We sectioned brains coronally at 25 µm on a vibratome (VT1200, Leica Biosystems, Illinois, USA). Three tissue series were obtained from each brain. We processed one for VT and TH, one for OT and 5‐HT, and one was kept spare.

### 2.2 Immunohistochemistry

All sections were incubated in a blocking solution consisting of 5% donkey serum (Sigma–Aldrich, St. Louis, Missouri, USA) and 0.2% Triton‐X‐100 (Sigma–Aldrich) in 1× PBS for 1 h at room temperature. Tissue sections from Series 1 were then incubated in blocking solution containing 0.2% guinea pig anti‐vasopressin antibody (T‐5048, BMA Biomedicals, Augst, Switzerland, AB_518680) and 0.1% sheep anti‐TH (NB300‐110, Novus Biologicals, Centennial, Colorado, USA, AB_10011104). Tissue sections from Series 2 were incubated in blocking solution containing 0.2% guinea pig anti‐OT (GP‐037‐50, Biosensis, Thebarton, Australia, AB_2492393) and 0.2% goat anti‐5‐HT (20079, ImmunoStar, Hudson, Wisconsin, USA, AB_572262). The sections were incubated in primary antibody solution for ∼60 h at 4°C and then rinsed in 1× PBS three times for 5 min each. Series 1 was then incubated in blocking solution containing 0.2% donkey anti‐sheep secondary antibody conjugated to Alexa Fluor 488 (ab150177, Abcam, Melbourne, Australia, AB_2801320) and 0.2% donkey anti‐guinea pig secondary antibody conjugated to Alexa Fluor 555 (706‐565‐148, Jackson ImmunoResearch, West Grove, Pennsylvania, USA, AB_3095467). Series 2 was incubated with 0.2% donkey anti‐goat secondary antibody conjugated to Alexa Fluor 488 (ab150129, Abcam, AB_2687506) and 0.2% donkey anti‐guinea pig conjugated to Alexa Fluor 555 (Jackson ImmunoResearch). Series were incubated in secondary antibody solution for 90 min at room temperature. The sections were then rinsed in PBS with 0.1% DAPI (4′,6‐diamidino‐2‐phenylindole) for 5 min and then rinsed in PBS for 5 min two more times. Finally, the sections were mounted onto gel‐coated slides and allowed to dry, then coverslipped with Fluoro‐Gel water‐based mounting medium (ProSciTech, Kirwan, Queensland, Australia) and sealed with clear nail polish.

For the *E. quoyii* brains, we initially tried an alternative anti‐OT antibody (T‐5021, Peninsular Laboratories, San Carlos, California, USA, AB_518526), but this failed to produce a positive signal in our sections. As such, we only report OT results from *L. whitii*. Because DA is a precursor to noradrenaline and adrenaline, we processed one spare *E. quoyii* brain series with sheep anti‐TH (as described above) and rabbit anti‐DA transporter (DAT) antibodies (ab184451, Abcam, AB_2890225). Primary and secondary antibody staining proceeded as described above, and we used a donkey anti‐rabbit secondary antibody conjugated to Alexa Fluor 647 (ab150075, Abcam, AB_2752244) to visualize the DAT immunosignal.

Sections were examined and images obtained using a Zeiss Axio Imager Z1 fluorescence light microscope and Zen PRO 3.8 software. We examined presence or absence of immunofluorescent cell bodies and fibers in each region directly at the microscope. We identified fibers as immunopositive structures with an elongated shape and no internal structural differentiation (such as the internal structural differentiation of a nucleus in a cell body). Fibers may or may not have irregular swollen varicosities as part of their structure. We did not systematically identify or quantify varicosities; however, they can be examined in the scanned slide images located at: https://doi.org/10.5281/zenodo.20341095. We took representative images of regions of interest at 20×, in a grid that was stitched together. All slides were also photographed using an Olympus Vs200 slide scanner (available at: https://doi.org/10.5281/zenodo.20341095). Brain regions were identified using a swift dragon (*Ctenophorus modestus*) atlas (Hoops et al. 2018) and verified using other available reptile brain atlases (Butler and Northcutt 1973; Cruce 1974; Del Corral et al. 1990; Díaz and Glover 2002; Greenberg 1982; Lopez et al. 1992; Medina et al. 1992; Northcutt 1967; Smeets et al. 1986a; ten Donkelaar 1998; ten Donkelaar et al. 1987). For brain regions we generally use abbreviations laid out in Hoops et al. (2018); see Table 2 for a full list.

**TABLE 1:** Brain regions displaying nonspecific binding of secondary antibodies. Anti‐guinea pig secondary antibodies were used on oxytocin and vasotocin antibodies, and anti‐sheep and anti‐goat secondaries were used for serotonin and tyrosine hydroxylase antibodies, respectively. C indicates cell bodies and f indicates fibers.

Brain region	Donkey anti‐guinea pig (Alexa Fluor 555)	Donkey anti‐goat (Alexa Fluor 488)	Donkey anti‐sheep (Alexa Fluor 488)
AS	C, f		C, f
VP	C, f		C, f
SD	C		C
mfb	f		f
IsM	C	C	
LL	C, f	C, f	C, f

**TABLE 2:** Abbreviations of brain region names.

Abbreviation	Brain region
3V	3rd ventricle
A8	Catecholaminergic cell group A8
ADVR	Anterior dorsal ventricular ridge
ac	Anterior commissure
Arc	Arcuate nucleus
AS	Anterior septum
BAC	Bed nucleus of the anterior commissure
BNST	Bed nucleus of the stria terminalis
CeL	Cerebellar nucleus (lateral part)
CeM	Cerebellar nucleus (medial part)
CG	Central gray
DLH	Dorsolateral hypothalamic nucleus
DMH	Dorsomedial hypothalamic nucleus
DMT	Dorsomedial thalamic nucleus
DS	Dorsal septum
DST	Dorsal striatum
Hb	Habenula
IP	Interpeduncular nucleus
IR	Inferior raphe
IS	Inferior septal nucleus
IsM	Isthmic nucleus (magnocellular part)
lfb	Lateral forebrain bundle
lfbv	Lateral forebrain bundle, ventral peduncle
LG	Lateral geniculate nucleus
LHA	Lateral hypothalamic area
LL	Lateral lemniscus
LS	Lateral septum
MA	Medial amygdala
mfb	Medial forebrain bundle
ML	Medial lemniscus
mlf	Medial longitudinal fasciculus
MT	Medial thalamic nucleus
NAcc	Nucleus accumbens
oc	Optic chiasm
ot	Optic tract
PaON	Paraventricular organ nucleus (formerly periventricular nucleus)
PL	Purkinje layer of the cerebellum
PM	Profound mesencephalic area
POA	Preoptic area
PVN	Paraventricular nucleus
RN	Red nucleus
SAT	Striatoamygdaloid transition area
SCH	Suprachiasmatic nucleus
SD	Supraoptic decussation
sm	Stria medullaris
SN	Substantia nigra
SON	Supraoptic nucleus
SR	Superior raphe
SRtF	Superior reticular field
STM	Bed nucleus of the stria terminalis (medial)
TSC	Torus semicircularis
VMH	Ventromedial hypothalamus
VMS	Ventromedial septal nucleus
VP	Ventral pallidum
VTA	Ventral tegmental area

### 2.3 Antibody Characterization

Controls consisted of processing sections as described above but without the primary antibodies. Four spare *L. whitii* brain series from two individuals were processed using only the secondary antibodies to verify that our results were not due to nonspecific staining of the secondary antibodies. All four control series were incubated with donkey anti‐guinea pig (conjugated Alexa Fluor 555) secondary antibody, used for both OT and VT. Two series were incubated with donkey anti‐goat secondary antibody (conjugated to Alexa Fluor 488), which was used for serotonin, and two series were incubated with donkey‐anti sheep secondary antibody (conjugated to Alexa Fluor 488), which was used for TH. Other than the omission of the primary antibodies, these control series were processed as described above. Slides were photographed using an Olympus Vs200 slide scanner and data viewed using Olympus OlyVIA 4.1.1 software.

## 3 Results

The distribution and density of immunoreactive (ir) perikarya and fibers were variable among species and individuals. All the regions in which we detected an immunopositive signal in each individual are listed in Table 3. Scans of all the slides we examined are available through our online repository for raw data located at: https://doi.org/10.5281/zenodo.20341095.

### 3.1 Antibody Characterization

In most instances of nonspecific binding, cell bodies and fibers were visibly bound to both secondary antibodies present. It is also worth noting that for all areas found to be fluorescent with only secondary antibodies, the qualitative level of fluorescence was far lower than that found in the experimental series processed with both primary and secondary antibodies.

**FIGURE 2:**
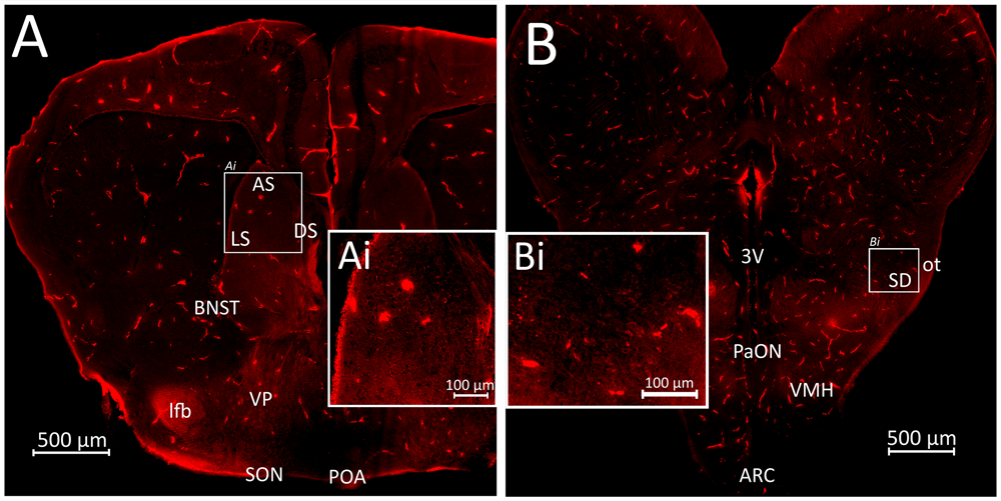
(A) Nonspecific binding of donkey anti‐guinea pig (Alexa Fluor 555) secondary antibody (used for imaging OT and VT immunoreactivity) in the anterior septum and lateral septum (Ai) and in the supraoptic decussation (Bi). In (B), this posterior portion of the supraoptic decussation may in fact be the posterior nucleus of the ventral supraoptic commissure. In both brain regions above, the same cells displayed nonspecific binding of the donkey anti‐sheep (Alexa Fluor 488) secondary antibody (used for imaging TH immunoreactivity).

The results of our control experiments are presented in Table 1. We found nonspecific binding of cell bodies and fibers in the anterior septum, and marginally in the lateral septum (LS) (Figure 2A). In the LS, we reported VT fibers in both *L. whitii* and *E. quoyii*, as well as OT fibers and perikarya in *L. whitii* and as such these results should be considered carefully. We also found nonspecific binding in the ventral pallidum, though very faintly. This region was found to have OT immunoreactivity in just one *L. whitii*, so this result should be viewed with caution. Consistent with the nonspecific binding found, these regions have not been found to contain OT or VT in other lizard species studied to date, though they are found in the LS in other taxa (e.g., Goodson et al. 2009; Olazábal and Sandberg 2020).

We found some nonspecific binding of secondary antibodies for VT, OT, and TH in cell bodies in the posterior part of the supraoptic decussation (SD) (Figure 2B). In our experimental brains, we found a great deal of immunoreactivity in this region across all signaling molecules and in both species studied. The nonspecific staining was weak, and was only in a very posterior part of the SD. It is unclear if these cell bodies are in fact in the SD or in the more posterior nucleus of the ventral supraoptic commissure. As such, we still report the immunoreactivity in the SD of our experimental brains below, but note that these results should be treated with caution.

We found fibers displaying nonspecific binding in the medial forebrain bundle for the VT, OT, and TH secondary antibodies. One *E. quoyii* was found to have VT‐ir here and so this is likely erroneous. We also found some cell bodies in the isthmic nucleus displaying nonspecific staining for OT, VT, and 5‐HT antibodies, though these were faintly stained. No immunoreactive cell bodies were found in this region in any brain slices and so this does not impact our results. For all secondary antibodies, we found nonspecific binding in the fibers of the lateral lemniscus. This area was found to have 5‐HT‐ir in *E. quoyii*. This pathway is involved in auditory processing (Gómez‐Martínez et al. 2023) and has not been found to contain 5‐HT immunoreactivity in other lizard species.

In addition to our control experiment, all our chosen primary antibodies (with the exception of the guinea pig anti‐OT; GP‐037‐50) have been successfully used in lizards previously (e.g., Hartline et al. 2017; Kabelik et al. 2014; Kabelik et al. 2022). The specificity of the anti‐vasopressin antibody (T‐5048) for VT and not OT has previously been determined in brown anoles, where preadsorption with OT eliminated OT‐immunoreactivity while leaving VT signal intact (Kabelik and Magruder 2014). This antibody has also previously been used to discriminate between VT and isotocin (an OT homolog) in plainfin midshipman fish (Goodson et al. 2003). Furthermore, the different distributions of VT and OT immunoreactivity we describe demonstrate the ability of these antibodies to differentiate between the two neuropeptides.

### 3.2 Vasotocin

**FIGURE 3:**
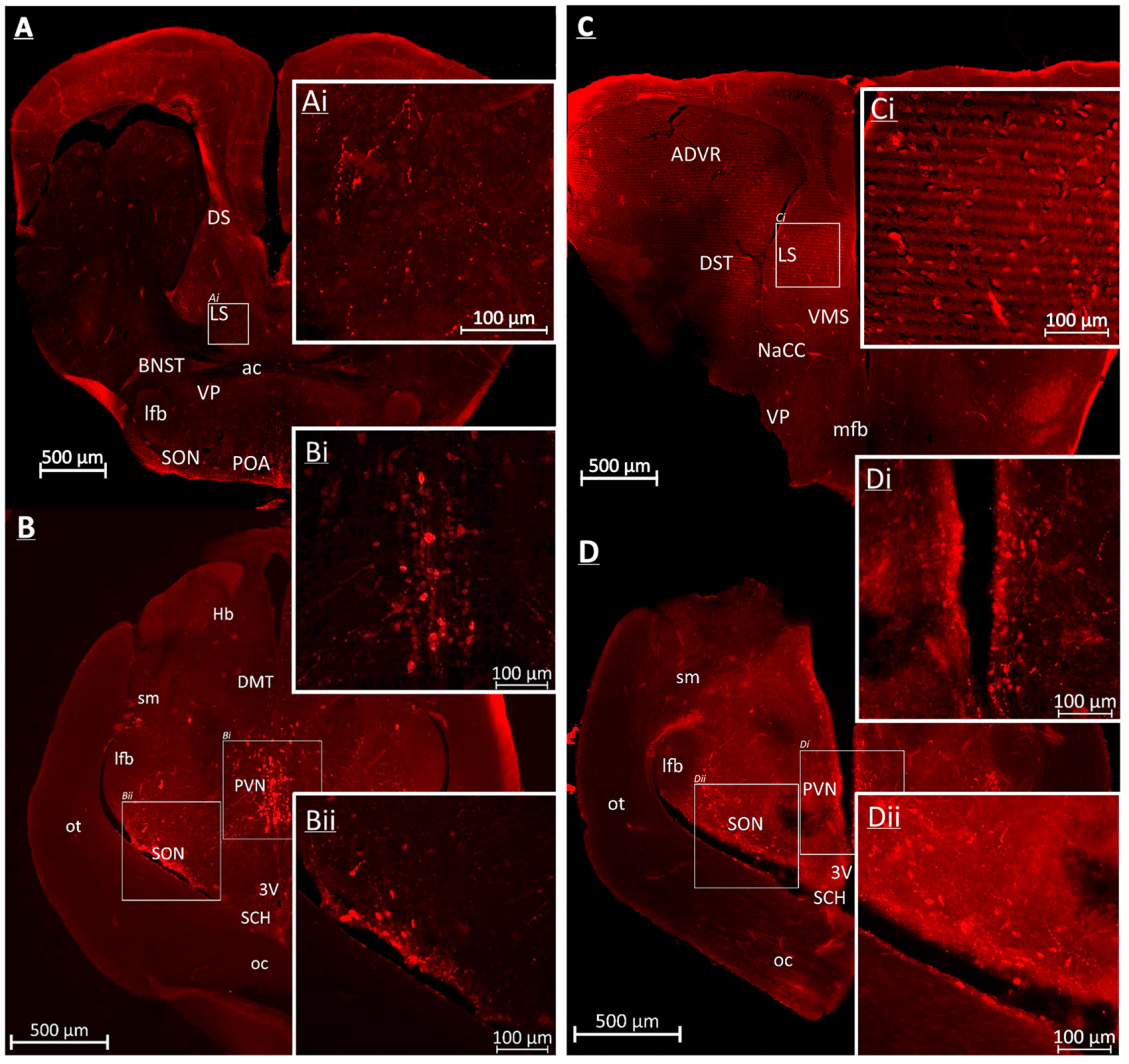
Representative images showing immunofluorescent labeling of vasotocin (VT) in the brains of (A and B) *E. quoyii* and (C and D) *L. whitii*. The lateral septum contained VT‐ir fibers in *E. quoyii*, visible here with large varicosities (Ai) but contained cell bodies in *L. whitii* (Ci). Cells and fibers in the PVN (Bi + Di) and the SON (Bii + Dii) were found in both species. See Table 2 for brain region abbreviations.

In both species we found VT‐ir cell bodies and fibers in the preoptic area (POA), supraoptic nucleus (SON), paraventricular nucleus (PVN), dorsomedial hypothalamus (DMH), paraventricular organ nucleus (PAoN), and SD (Figure 3). In both species we also found a highly immunoreactive population of VT‐ir cell bodies and fibers in the area dorsal to the SON and ventral to the stria medullaris (sm), pressed against the edge of the lateral forebrain bundle (lfb) (Figure 3D). There is no well‐defined brain region here, and so we refer to this area as the lfb boundary.

In both species we found VT‐ir fibers in the bed nucleus of the anterior commissure (BAC), medial amygdala (MA), bed nucleus of the stria terminalis (BNST), arcuate nucleus (Arc), and the ventromedial hypothalamus (VMH). Dense fibers in the Arc are oriented laterally toward the SD. Another dense network of VT‐ir fibers is oriented ventrolaterally from the PVN, toward the SON. We found dense fibers in the suprachiasmatic nucleus (SCH) of both species, and one *E. quoyii* individual had a small number of VT‐ir cell bodies here. In *E. quoyii* we found fibers in the LS, but we found perikarya in the same region as well in *L. whitii* (Figure 3Ai + Ci). However, our control slides showed nonspecific binding of antibodies to cells in the nearby anterior septum and potentially marginally in the LS itself (Figure 2A) and so this finding should be treated with caution. Although our study does not distinguish between bypassing fibers or fiber terminals, both axons and dendrites have been shown to release VT along their length (Morris and Pow 1991), and so any process detected likely represents vasotocinergic activity in a given region.

In *L. whitii*, we found additional cell bodies in the dorsomedial thalamic nucleus (DMT) and the central gray, as well as in the ventral tegmental area (VTA) and superior raphe, which are dopaminergic and serotonergic regions, respectively. VT‐ir fibers were also found in the inferior septal nucleus (IS) and the optic chiasm.

### 3.3 Oxytocin

**FIGURE 4:**
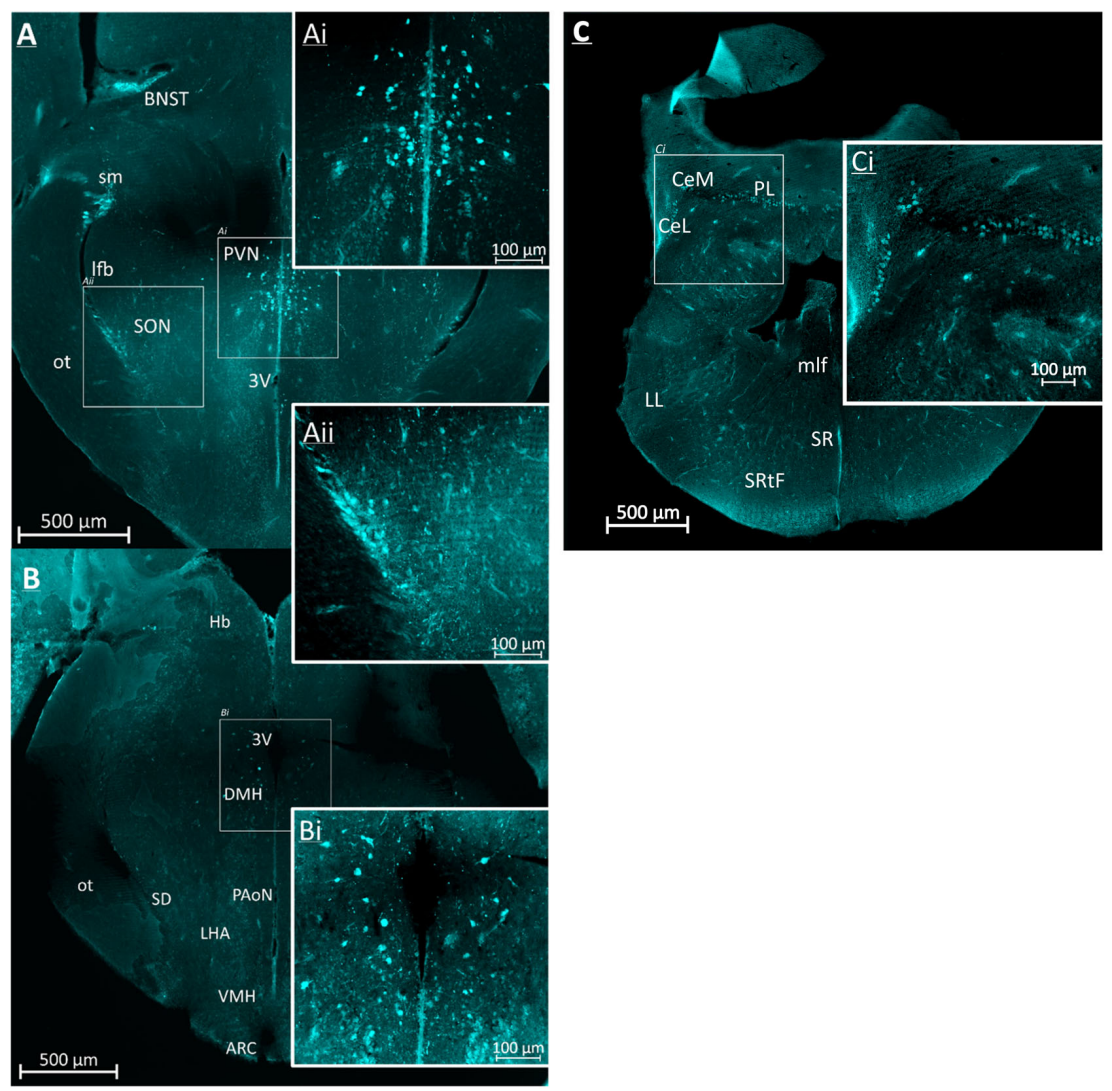
Representative images showing immunofluorescent labeling of oxytocin (OT) in the brain of *L. whitii*. Insets show the dense groups of perikarya and fibers in the PVN (Ai) and SON (Aii). (Bi) Signal in the DMH is shown. (Ci) The novel groups of OT‐ir cell bodies in the cerebellum are shown. See Table 2 for brain region abbreviations.

We only examined OT in the brains of *L. whitii*. We found OT‐ir cell bodies and fibers in the LS, POA, SON, SCH, DMT, DMH, PAoN, lateral hypothalamic area (LHA), SD, VMH, central gray, medial longitudinal fasciculus (mlf), superior raphe, and superior reticular field (SRtF) (Figure 4). In all specimens we found numerous cell bodies and fibers in the same uncharacterized area at the lfb boundary where we observed VT‐ir cell bodies and fibers. This area seems to bridge the region from the SON to the PVN and sm/BNST (Figure 4A). In one specimen, we found a densely populated layer of OT‐ir perikarya in the cerebellar nuclei (CeL and CeM) and Purkinje layer of the cerebellum (PL) (Figure 4C). Dense OT‐ir fiber populations were found in the MA and Arc, and fibers were also found in the nucleus accumbens (NAcc), optic chiasm, and VTA.

### 3.4 Tyrosine Hydroxylase

**FIGURE 5:**
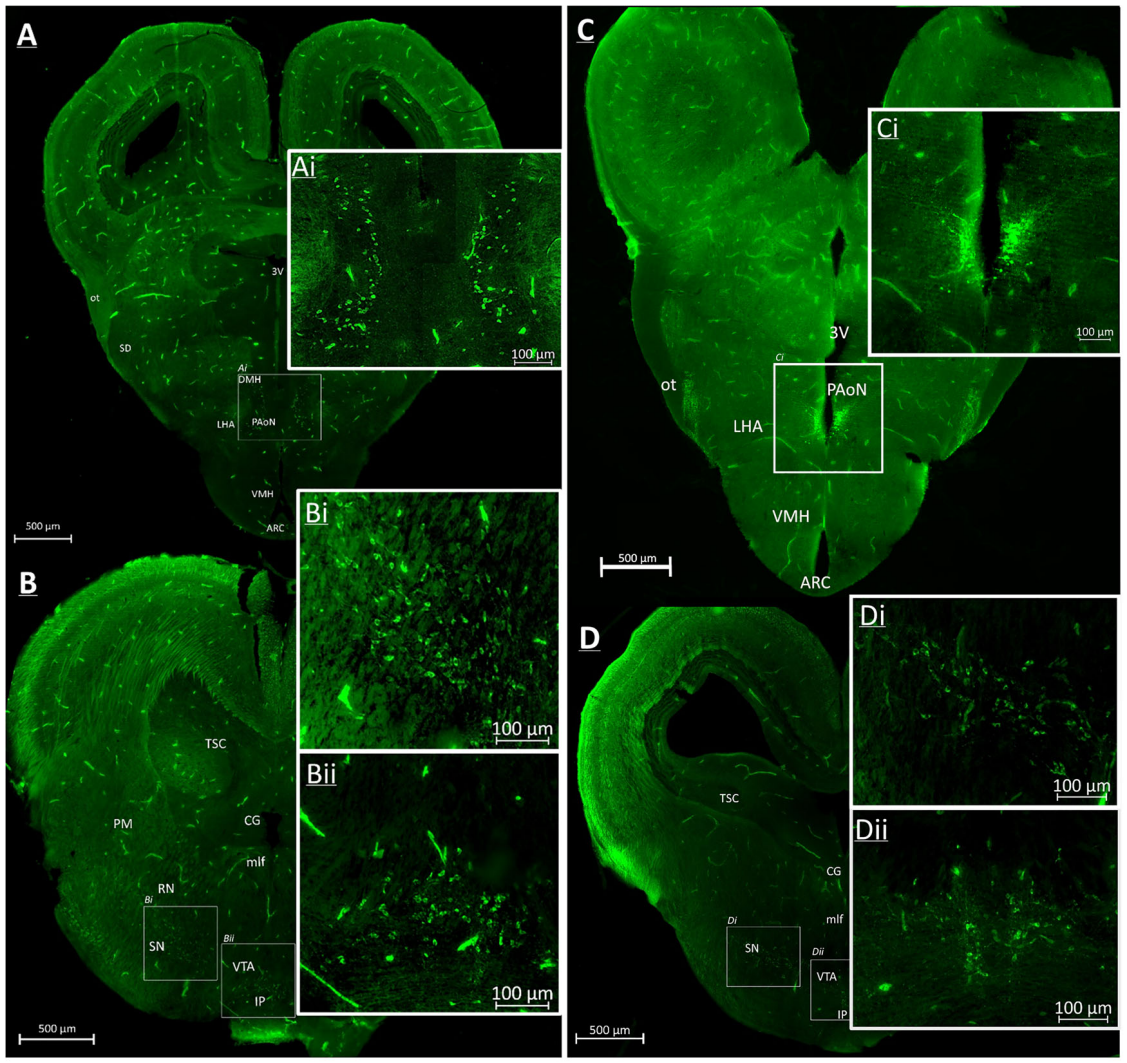
Representative images showing immunofluorescent labeling of tyrosine hydroxylase in the brains of (A and B) *E. quoyii* and (C and D) *L. whitii*. The PAoN and DMH of both species displayed immunoreactivity (Ai + Ci). Similarly, cells and fibers in the SN (Bi + Di) and the VTA (Bii + Dii) were common to both species. See Table 2 for brain region abbreviations.

In both *E. quoyii* and *L. whitii*, we found TH‐ir perikarya in the SCH, PAoN, DMH, substantia nigra, and VTA (Figure 5). In one *E. quoyii* individual, we found a weakly immunopositive layer of TH‐ir cell bodies at the boundary between the torus semicircularis and the cerebellum, which we did not observe in any *L. whitii*. Otherwise, all areas showing signal in *E. quoyii* were found in *L. whitii*. By contrast, we found several areas uniquely immunopositive in *L. whitii*, including cell bodies and fibers in the LHA, interpeduncular nucleus, superior raphe, and catecholaminergic cell group A8. We also found TH‐ir fibers in the lfb boundary, both medially, oriented toward the POA, and laterally, pressed between the lfb and the optic tract.

**FIGURE 6:**
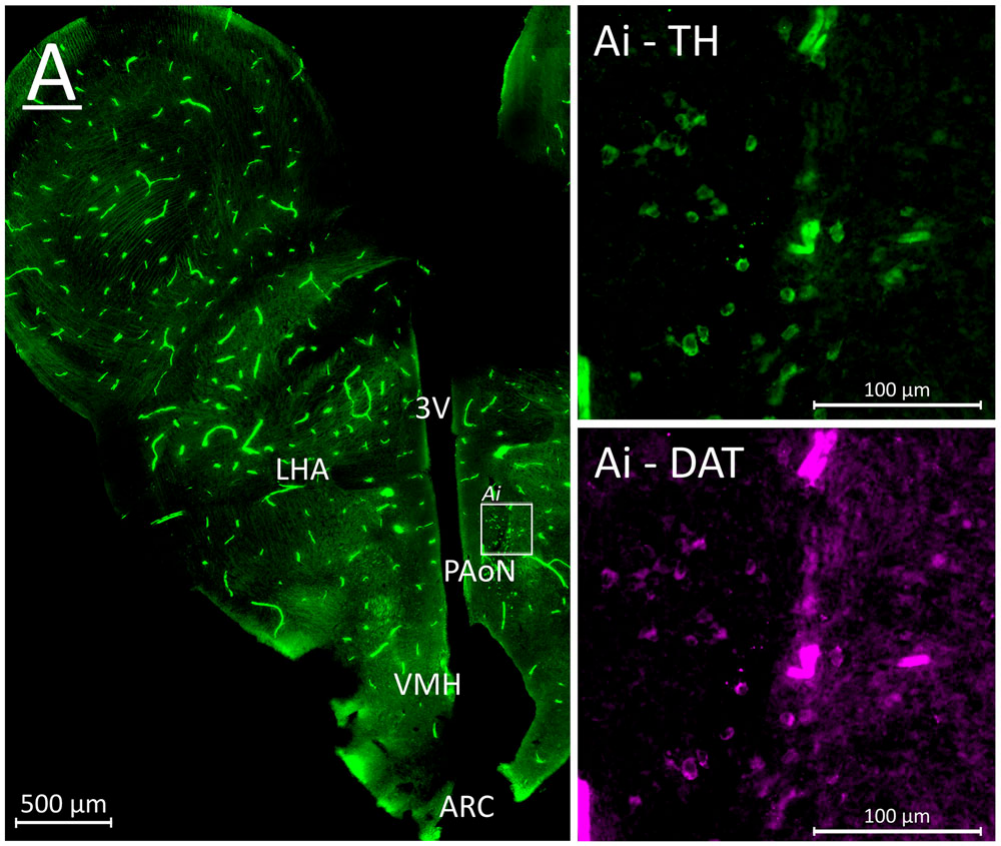
Colocalization of tyrosine hydroxylase (TH) and dopamine active transporter (DAT) in the PaoN of *E. quoyii*.

In an additional *E. quoyii* brain series we colocalized for TH and the DA active transporter (DAT) to verify that the TH‐immunopositive regions were dopaminergic, we found cell bodies showing positive signal for both DAT and TH in the PAoN, the VTA, and the central gray (Figure 6). However, the DAT signal was weak, and so where we did not find DAT/TH colocalization, we do not necessarily rule out dopaminergic populations (e.g., the substantia nigra did not show DAT immunoreactivity despite being one of the primary DA synthesizing regions) (Felten and Sladek 1983; Lopez et al. 1992).

### 3.5 Serotonin

**FIGURE 7:**
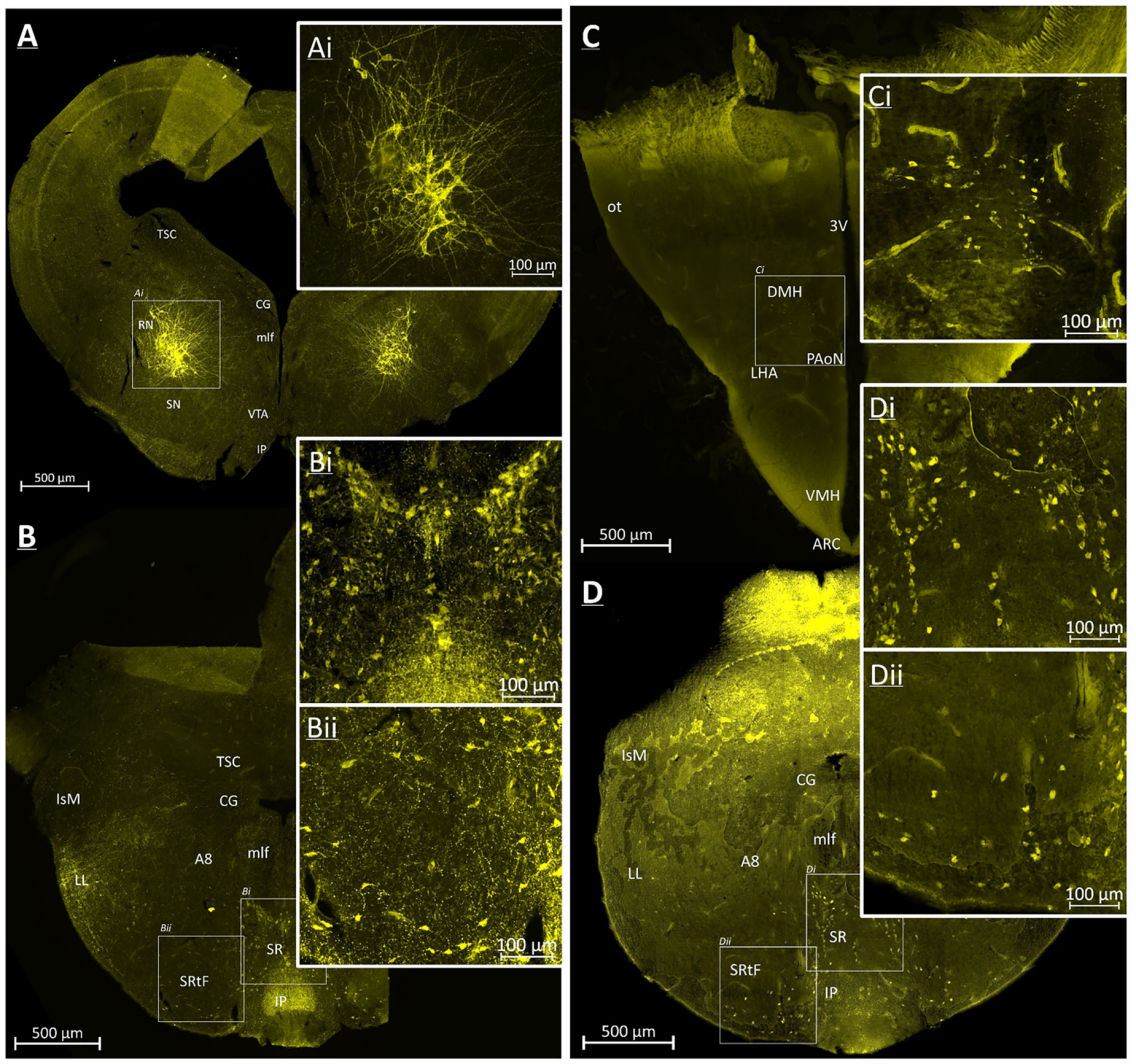
Representative images showing immunofluorescent labeling of serotonin in the brains of (A and B) *E. quoyii* and (C and D) *L. whitii*. Insets show immunopositive signal in the red nucleus of *E. quoyii* (Ai), the DMH of *L. whitii* (Ci), and cells and fibers in the superior raphe (Bi + Di) and the superior reticular field (Bii + Dii) in both species. See Table 2 for brain region abbreviations.

Both *E. quoyii* and *L. whitii* had serotoninergic cell bodies in the PAoN, the superior raphe, and the SRtF (Figure 7). In both species, we found 5‐HT‐ir fibers but no cell bodies in the habenula, the SD, the central gray, and the medial lemniscus. Both species also had fibers in the interpeduncular nucleus, but only *E. quoyii* showed expression on cell bodies here. We also found a dense population of cell bodies and fibers in the red nucleus of *E. quoyii* (Figure 7A). In *L. whitii* we found additional cell bodies and fibers in the SCH, the DMH (Figure 7C), LHA, and inferior raphe, as well as fibers in the VMH and cerebellar nuclei.

## 4 Discussion

We report numerous interspecies differences (and similarities) in the distribution of immunoreactive cell bodies and fibers between *L. whitii* and *E. quoyii*. Across the three neural markers in which comparisons were made (VT, TH, and 5‐HT), *L. whitii* tended to show immunopositive signal across a larger number of brain regions compared to *E. quoyii*. We also found that in the case of VT, *L. whitii* had more immunoreactivity in areas that are usually more associated with DA and 5‐HT. This general increase in the presence of these neurotransmitters in *L. whitii* suggests that they may play a role in social behavior similar to that seen in other taxa (Adkins‐Regan 2009; Bacqué‐Cazenave et al. 2020), and highlights neurobiological differences that may be linked to the more social life of *L. whitii* relative to *E. quoyii*. However, there were several regions with no signal in *L. whitii* that showed immunoreactivity in *E. quoyii*, such as the strong 5‐HT presence in the red nucleus, and the TH‐ir cell bodies in the torus semicircularis. In the case of OT, no comparison could be made to *E. quoyii*, but we did find OT in brain regions of *L. whitii* previously unreported in reptiles: the DMH and cerebellum. We draw comparisons with the literature on previously studied lizards. For more detailed info, see extended data tables detailing the distribution of immunoreactive cells and fibers in other lizards, for VT (Table S1), OT (Table S2), TH (Table S3), and serotonin (Table S4) at our raw data depository at: 10.5281/zenodo.20341095.

### 4.1 Vasotocin

In both *E. quoyii* and *L. whitii*, VT immunoreactivity was found in the POA, SON, PVN, DMH, PAoN, and SD. Numerous fibers were also found throughout the hypothalamic regions, and these patterns generally match those found in other vertebrates (reviewed in Wilczynski et al. 2017). In all lizards studied to date, including here, VT‐ir perikarya have been found most consistently in the SON and PVN (Goossens et al. 1979; Kabelik et al. 2008), as well as in the POA (Bons 1983; Hillsman et al. 2007; Kabelik et al. 2013). In addition to these areas, VT‐ir cells and fibers have been found in the lfb, PaON, and VMH of *Anolis carolinensis* (Kabelik et al. 2022; Propper et al. 1992), all of which express VT immunoreactivity in the two skinks studied here.

In contrast, we found VT‐ir cell bodies and fibers in the DMH of both *L. whitii* and *E. quoyii*, which has not been reported in other lizards. The DMH has been found to contain VT‐ir cell bodies in the turtle *Psuedemys scripta* (Smeets et al. 1990), as well as in mammals (Caffé and van Leeuwen 1983). In mammals, VT signaling in the DMH is involved in controlling neuroendocrine circadian rhythms (Kalsbeek et al. 2008, 2010) and it would be unsurprising if it played a similar role in skinks, although why vasotocinergic cells are not found here in other lizards remains unclear.

Finally, we found VT‐ir perikarya and fibers in the SD of both species. VT‐ir cell bodies have been found in the same region in the snake *Python regius* (Smeets et al. 1990) and the mouse *Mus musculus* (Rood and De Vries 2011). In other lizards studied to date, no VT‐ir has been reported in the SD, though it is possible that immunoreactivity in this region has been recorded as posterior sections of the SON as opposed to SD given the proximity of these regions (Hoops et al. 2018). It should be noted that some evidence of nonspecific binding of secondary antibodies was found in the posterior part of the SD, and therefore these results should be considered cautiously (Table 1).

In *L. whitii*, we found VT‐ir fibers and cell bodies in the 5‐HT‐producing raphe nuclei and the DA‐producing VTA. In mice, social interactions activate VT receptors in the raphe nuclei, potentially to release 5‐HT and mediate prosocial responses (Patel et al. 2022), and in the VTA of Syrian hamsters, OT mediates DA release to promote social behavior (Song et al. 2016). It is possible that the observed VT cells in these hindbrain regions fulfill a similar function in *L. whitii*. The lack of VT‐ir in these regions in *E. quoyii* suggests that the differences in social systems may be underpinned by greater connectivity between neurotransmitter systems.

### 4.2 Oxytocin

OT immunoreactivity in *L. whitii* was found in the POA, SON, PVN, DMH, VMH, and SD, a distribution broadly consistent with that seen in other vertebrates (Knobloch et al. 2014) (but see above for potential nonspecific binding in the SD). Indeed, all lizard species studied to date show OT‐ir cell bodies in the PVN and SON (Goossens et al. 1979; Kabelik and Magruder 2014; Thepen et al. 1987), with additional populations in the VMH and POA found in some species (Bons 1983).

OT‐ir cell bodies and fibers were also found in the DMH of *L. whitii*, which has been previously reported in mammals (Shi and Bartness 2000), but not in other lizards. Stimulation of the DMH in rats is involved in the milk‐ejection reflex, via OT release in the SON (Honda and Higuchi 2007). Whether this population of OT receptors has a maternal role in *L. whitii* requires further research. In the hindbrain we found OT‐ir cell bodies in posterior areas such as the mlf, raphe nuclei, and reticular formations, which have not been reported in other lizards. OT is known to modulate serotonin release in these regions in mammals (Oubraim et al. 2023) and its function may be similar here.

In one *L. whitii*, we found a population of OT‐ir cells in the medial and lateral cerebellar nuclei, as well as in the Purkinje layer of the cerebellum. These cell bodies displayed weak signal but were numerous and densely packed. OT has previously been found elsewhere in the cerebellum of rats and mice, although its functional role here remains unclear (Li et al. 2024). To our knowledge, OT has not yet been reported in the cerebellum of reptiles.

For many of the regions described here, only one out of the four *L. whitii* individuals expressed immunoreactive cell bodies or fibers, but it was not always the same individuals, suggesting true individual difference as opposed to differences in antibody sensitivity. For the specific expression patterns we observed in each individual, see Table 3.

**TABLE 3:** Individuals displaying immunopositive fibers or perikarya for vasotocin, oxytocin, tyrosine hydroxylase, and serotonin. EQ = *Eulamprus quoyii*, LW = *Liopholis whitii*. Note that no *E. quoyii* were processed for oxytocin. Table is organized by brain region, with anterior regions first and more posterior regions further down the table. See Table 2 for brain region abbreviations. We have marked entries in this table with an asterisk if nonspecific binding of the relevant secondary antibody was found in the control experiment in the same region, indicating results that should be treated with caution. We found nonspecific binding in the anterior septum, the ventral pallidum, the supraoptic decussation, the medial isthmic nucleus, the medial fiber bundle, and the lateral lemniscus (Table 1).

Brain region	Vasotocin		Oxytocin		Tyrosine hydroxylase		Serotonin	
	Fibers	Perikarya	Fibers	Perikarya	Fibers	Perikarya	Fibers	Perikarya
Nacc	EQ2	—	LW95	—	—	—	—	—
LS	EQ2	LW95	LW54	LW146	—	—	—	—
VP	—	—	LW54*	—	—	—	—	—
VMS	EQ3	—	—	—	—	—	—	—
mfb	EQ3*	—	—	—	LW133*	—	LW95	—
DS	—	—	—	—	—	—	EQ1	—
IS	LW54 LW133	—	—	—	—	—	—	—
ac	EQ2 LW133	—	—	—	LW54	—	—	—
SAT	EQ1 EQ3	—	—	—	—	—	—	—
POA	EQ1 EQ2 EQ3 LW54 LW133 LW146	EQ2 LW95	LW54 LW95 LW133 LW146	LW95	—	—	—	—
SON	EQ1 EQ2 EQ3 LW54 LW133 LW46	EQ1 EQ2 EQ3 LW54 LW133 LW46	LW54 LW95 LW133 LW146	LW54 LW95 LW133 LW146	—	—	—	—
BAC	EQ1 EQ2 LW54 LW146	—	LW95	—	—	—	—	—
MA	EQ2 LW54 LW95 LW133 LW146	—	LW54 LW95 LW146	—	—	—	—	—
BNST	EQ1 EQ2 EQ3 LW54 LW133 LW146	—	LW54 LW95 LW133 LW146	LW54	—	—	LW95	—
STM	—	—	—	—	—	—	—	—
lfb boundary	EQ1 EQ2 EQ3 LW133 LW146	EQ1 EQ2 EQ3 LW54 LW133 LW46	LW54 LW95 LW133 LW146	LW54 LW95 LW133 LW146	LW54 LW133	—	—	—
sm	—	—	LW95	LW95	—	—	—	—
PVN	EQ1 EQ2 EQ3 LW54 LW95 LW133 LW146	EQ1 EQ2 EQ3 LW54 LW95 LW133 LW146	LW54 LW95 LW133 LW146	LW54 LW95 LW133 LW146	—	—	—	—
SCH	EQ1 EQ2 EQ3 LW146	EQ1	LW95 LW133 LW146	LW133	LW54	EQ2 EQ3 LW54 LW95 LW146	LW133	LW133
oc	LW54 LW146	—	LW146	—	—	—	EQ1	—
Hb	EQ3	—	—	—	LW133	—	EQ1 EQ3 LW95	—
DMT	LW133	LW133	LW95	LW95	—	—	—	—
LG	—	—	—	—	—	—	EQ1	—
lfbv	—	—	LW54	—	—	—	—	—
MT	EQ2	—	—	—	—	—	—	—
DMH	EQ2 EQ3 LW54 LW95 LW133 LW146	EQ2 EQ3 LW54 LW95 LW133 LW146	LW54 LW95 LW133 LW146	LW54 LW133 LW146	LW95 LW146	EQ2 LW95 LW146	LW95 LW133	LW133
DLH	LW133	—	—	—	—	—	—	—
PaON	EQ2 LW54 LW133 LW46	EQ2 LW54 LW133 LW46	LW54 LW146	LW146	EQ2 EQ3 LW54 LW95 LW133 LW146	EQ2 EQ3 LW54 LW95 LW133 LW146	EQ1 EQ2 EQ3 LW54 LW95 LW146	EQ1 EQ2 EQ3 LW54 LW146
LHA	LW54	—	LW54 LW95 LW133	LW133	LW146	LW95 LW146	LW95 LW133	LW133
SD	EQ1* EQ2* EQ3* LW54* LW95* LW133*LW146*	EQ1* EQ2* EQ3* LW133* LW146*	LW54* LW95* LW133*LW146*	LW54* LW95* LW146*	LW133*	—	EQ1 EQ2 LW54 LW95	—
VMH	EQ1 EQ2 EQ3 LW54 LW133 LW46	—	LW54 LW95 LW146	LW146	LW133 LW146	LW146	LW95 LW146	—
Arc	EQ1 EQ2 EQ3 LW54 LW95 LW133 LW146	—	LW54 LW95 LW133 LW146	—	—	—	LW95	—
CG	LW146	LW133	LW95 LW133	LW95 LW146	LW133	EQ2	EQ1 LW95	—
TSC	—	—	—	—	—	EQ3	—	—
PM	—	—	LW95	—	LW133	—	—	—
mlf	—	—	—	LW95 LW133	LW133	—	—	—
RN	—	—	—	—	—	—	EQ1 EQ2	EQ1 EQ2
SN	—	—	—	—	EQ2 EQ3 LW95 LW146	EQ2 EQ3 LW95 LW146	—	—
VTA	LW54	LW54 LW95	LW95	—	EQ1 EQ2 EQ3 LW54 LW95 LW133 LW146	EQ1 EQ2 EQ3 LW54 LW95 LW133 LW146	—	—
IP	—	—	—	—	LW95	LW95	EQ1 LW146	EQ3
IsM	LW54*	—	—	—	—	—	—	—
A8	—	—	—	—	LW133	LW133	—	—
LL	—	—	—	—	—	—	EQ1* EQ2*	—
ML	LW54	—	LW146	—	—	—	EQ1 EQ2 LW95	—
SR	LW54	LW54 LW95	—	LW146	LW133	LW133	EQ1 EQ2 LW95 LW146	EQ1 EQ2 EQ3 LW95 LW146
SRtF	LW133	—	LW133	LW133 LW146	—	—	EQ1 EQ2 LW54 LW95 LW133 LW146	EQ1 EQ2 EQ3 LW54 LW95 LW133 LW146
CeM	LW133	—	LW95; LW146	LW95 LW146	—	—	LW95	—
CeL	LW133	—	LW95 LW146	LW95	—	—	LW95	—
PL	—	—	LW95	LW95	—	—	—	—
IR	—	—	—	—	—	—	LW54 LW95	LW54 LW95

In both species, we found VT and OT‐ir cell bodies and fibers in the lfb boundary area. Fibers were oriented dorsoventrally, starting from the SON, skirted around the lfb, and joined with fibers in the sm and the MA. These fibers passed on both the medial and lateral sides of the lfb, the latter being sandwiched between the lfb and optic tracts. Cell bodies were also present here, but not in the densities they were found in the nearby SON and PVN. Similar populations of VT and OT‐ir cell bodies and fibers have been reported in other lizards, snakes, and turtles (Fernández‐Llebrez et al. 1988; Kabelik et al. 2008; Bjørnebekk et al. 2013; Kabelik and Magruder 2014; Propper et al. 1992; Silveira et al. 2002; Smeets et al. 1990; Stoll and Voorn 1985), although often mentioned only in passing and it is unclear whether these cells and fibers are distinct subregions or perhaps are a dorsal extension of the SON. If this area does represent a distinct oxy‐ and vasotocinergic region, its functional role and connections to other areas warrant further study.

### 4.3 Tyrosine Hydroxylase

We found TH immunoreactivity in the SCH, DMH, PAoN, SN, and VTA in both species, broadly matching that found in other reptiles (Kabelik et al. 2014; Lopez et al. 1992; Smeets et al. 2001; Wolters et al. 1984; Woolley et al. 2004). Beyond these regions, we observed that *L. whitii* had TH‐ir cell bodies distributed across a wider number of brain areas compared to *E. quoyii*. For example, we found TH‐ir cell bodies in the LHA of *L. whitii*, but not *E. quoyii*. Whether this is linked to the differences in social behavior is unclear. DA activity in this region is linked to learning of stimuli relevant to important events, such as social interactions, but also food, water, and pain (Sharpe 2024); stimuli that are presumably important to both social and nonsocial lizards. Indeed, TH‐ir has been found here in several other non‐grouping lizards (Kabelik et al. 2014; Lopez et al. 1992; Woolley et al. 2004). Therefore, the reason for a lack of immunoreactivity in the *E. quoyii* is unclear. Only *L. whitii* displayed TH‐ir cells and fibers in the VMH. Green anoles have TH‐ir cell bodies in the VMH, but the closely related brown anole does not (Kabelik et al. 2014; Lopez et al. 1992), suggesting that this area of the brain may show particularly high variability between species, unlike the well‐conserved TH‐ir expression in the VTA and SN.

Because TH is a rate‐limiting enzyme involved in catecholamine production in general, we cannot say for certain whether the regions identified here are dopaminergic or noradrenergic. However, a study on *Gekko gecko* using a specific DA antibody found DA‐ir cell bodies in the SCH, PAoN, VTA, and SN (Smeets et al. 1986b), all of which showed signal for TH in the present study. Although DA is a precursor of noradrenaline, Smeets et al. report only “a light staining” of perikarya in the locus coeruleus, the principal source of noradrenaline in the brain, compared to “very intense staining” in dopaminergic regions. We did not detect any TH immunoreactivity in the locus coeruleus in either skink species, suggesting our results reflect dopaminergic signal.

To verify the dopaminergic nature of our TH immunoreactivity we co‐localized TH with a DAT antibody in *E. quoyii* brain sections. Although DAT antibodies do not produce as robust a signal as TH antibodies, we were able to confirm that TH‐ir perikarya in the PAoN, VTA, and central gray were also immunopositive for DAT. Additionally, previous work has shown that noradrenergic regions in *A. carolinensis* are limited to the locus coeruleus, SRtf, A5 cell group, laminar nucleus, ventromedial nucleus, nucleus of the solitary tract, area postrema, and vagal motor nucleus (Lopez et al. 1992), none of which showed immunoreactivity for TH in our study. We are therefore cautiously confident that we have primarily described patterns of DA immunoreactivity throughout the skink brains, although future work should investigate this further.

### 4.4 Serotonin

In both *E. quoyii* and *L. whitii*, we found 5‐HT signal most consistently in the raphe nuclei and SRtF, which is expected as these are the brain's principal serotonergic regions (Ayala‐Guerrero et al. 1991; Bennis et al. 1990; Hartline et al. 2017). We also found 5‐HT‐ir perikarya in the PAoN, which has previously been found in other lizards such as *Varanus exanthematicus* and *Gekko gecko* as well as in the interpeduncular nucleus, where we detected 5‐HT in some individuals (Smeets and Steinbusch 1988; Wolters et al. 1985). Similar distributions of 5‐HT have been found in amphibians (Clairambault et al. 1994), birds (Challet et al. 1996), and mammals (Steinbusch 1981).

One *L. whitii* had additional 5‐HT‐ir cell bodies in the SCH, DMH, and LHA, and in all instances these exact cells also showed OT‐ir. This could indicate cross reactivity of the antibodies, but the same pattern was not found in every OT‐ir brain region in this individual. There is a great deal of interaction between the serotonergic and oxytocinergic systems, and in mammals, 5‐HT producing cells in the raphe nuclei project to the PVN where they modulate OT release and control affiliative behavior between mothers and offspring (Liu et al. 2023). The presence of 5‐HT in these hypothalamic areas in *L. whitii* may hint at similar pathways controlling maternal affiliation in skinks.

We also found a dense group of 5‐HT cell bodies and fibers in the red nucleus of *E. quoyii*, an area primarily involved in controlling motor functions (Basile et al. 2020). No signal was found here in *L. whitii*, and this has not been reported in other reptiles to our knowledge. Note that 5‐HT is a known neuromodulator of this region in rats (Licata et al. 2001), and possibly plays a similar role in *E. quoyii*, although its absence in other lizards requires further study.

### 4.5 Within‐Species Variation

Across all four transmitters of interest, there was substantial interindividual variation in their distribution, with many brain regions only showing a signal in one individual (Table 3). In many instances, such signals were relatively weak, and so similar groups of cells and fibers were likely present in other individuals, but below our detectable threshold. However, these differences could also reflect variability in antibody uptake or true individual variation, and a quantitative study with a larger number of subjects could tease apart these potential explanations. Although individual variation is well established in animal behavior, the neural mechanisms underlying such variation remains poorly understood (Wilson et al. 2019). Several studies in humans have linked personality differences to large‐scale structural variation in the brain such as cortical thickness or volume (e.g., Bjørnebekk et al. 2013; Schilling et al. 2012), but rarely have animal models been leveraged to look at finer scale variation in neural architecture, and whether this correlates to behavior (but see Linneweber et al. 2020). There is substantial individual variation in sociality and parental tolerance in *L. whitii* (While et al. 2009), which could partly be the result of interindividual differences in neural signaling involving the neurotransmitters examined here.

### 4.6 Future Directions

Future work should aim to compare the distribution of OT between these two species, as well as others that vary in sociality, as this has the potential to be particularly informative with regards to the differences in social behavior. For instance, a high density of OT receptors in the NAcc predicts stronger parental and social bonds in rodents (Olazábal and Sandberg 2020), and similarly, higher density of OT receptors in the LS predicts gregariousness in estrildid finches (Goodson et al. 2009). In *L. whitii*, small groups of OT‐ir fibers were found in the LS and NAcc, but unfortunately comparisons to *E. quoyii* could not be made.

We also did not investigate the distribution of neurotransmitters in the main and accessory olfactory bulbs as they are separate from the rest of the brain in lizards and difficult to dissect from the skull (Hoops 2015). However, chemical communication is important for many lizards (Mason and Parker 2010) and these areas are known to play a role in social behavior (Keller et al. 2009). The accessory olfactory bulbs have been shown to differ in morphology between two closely related rodents that differ in social ecology (Fernández‐Aburto et al. 2020) and we may expect similar patterns to be present in skinks.

## 5 Conclusions

Here we show substantial inter‐ and intraspecific differences in the distribution of key social neurotransmitters. In doing so, these results provide potential insights into the mechanisms accompanying major evolutionary transitions in sociality. For example, 5‐HT and DA activity in forebrain regions have been hypothesized to underpin the prosocial shift in behavior that accompanied the evolution of great apes and hominids, namely, lower intrasocial aggression, increased cooperation, and social monogamy (Raghanti et al. 2018). Likewise, increased OT and VT activity in the forebrain has been linked to monogamy in voles (Insel and Shaprio 1992; Insel et al. 1994), and social tolerance in songbirds (Goodson and Wang 2006; Goodson et al. 2009). The social system of *L. whitii* is characterized by social monogamy and decreased aggression toward offspring (Chapple and Keogh 2005; While et al. 2009), and our observations on the distributions of VT, OT, DA, and 5‐HT generally hint toward an increase of these neurotransmitters in the brains of this family‐living species compared to *E. quoyii*. To test this idea further, we need data on neurotransmitter distribution and abundance across a greater number of species. The Tiliquini provide an excellent model system for such an approach as they include species that span the entire spectrum of sociality (Chapple 2003; Whiting and While 2017, 2026). Further work should take advantage of this system by comparing the neuroanatomy of representative members of this group, allowing us to strengthen (or challenge) the inferences made here. Moreover, because tiliquin skinks represent a wholly independent transition to social grouping (Van Dyke et al. 2021), identifying patterns in social skinks that parallel those found in other social vertebrates would provide further evidence for convergent mechanisms underpinning the evolution of complex sociality.

## Supporting information




**Supporting Information**: cne70184‐sup‐0001‐TableS1‐S4.xlsx

## Data Availability

The data that support the findings of this study are available at: https://doi.org/10.5281/zenodo.20341095. This openly accessible data repository contains all of the slide scanned images, as well as data Tables S1–S4 detailing the distribution of immunoreactive cells and fibers in other lizards.
